# Interactions Between Non-Coding RNAs and HIF-1alpha in the Context of Colorectal Cancer

**DOI:** 10.3390/biom15040510

**Published:** 2025-04-01

**Authors:** Lianfeng Gong, Haixia Zhang, Ying Liu, Xianwang Wang, Ruohan Xia

**Affiliations:** School of Basic Medicine, Health Science Center, Yangtze University, Jingzhou 434023, China; 2022710991@yangtzeu.edu.cn (L.G.); 2022711003@yangtzeu.edu.cn (H.Z.); 2021710973@yangtzeu.edu.cn (Y.L.)

**Keywords:** non-coding RNA, hypoxia-inducible factor-1 alpha, colorectal cancer, clinical implications

## Abstract

Hypoxia-inducible factor-1α (HIF-1α), a master regulator of cellular adaptation to hypoxia, drives colorectal cancer (CRC) progression by fueling angiogenesis, metastasis, and therapy resistance. Emerging evidence delineates intricate crosstalk between non-coding RNAs (ncRNAs)—including microRNAs, long non-coding RNAs, and circular RNAs—and HIF-1α, forming bidirectional regulatory networks that orchestrate CRC pathogenesis. By interacting with HIF-1α, these non-coding RNAs contribute to the orchestration of the aggressive hypoxic tumor microenvironment. Recent studies have evaluated the clinical potential of lncRNAs and miRNAs in the realms of non-invasive liquid biopsies and RNA-targeted therapies. This review offers a comprehensive synthesis of recent investigations into the mechanisms by which lncRNAs and miRNAs interact with HIF-1α to modulate CRC progression. Additionally, we further explore the clinical implications of ncRNA/HIF-1α crosstalk, emphasizing their potential as diagnostic biomarkers and therapeutic targets, while also spotlighting intriguing and promising areas of ncRNA research. Methods: In this study, our search strategy employed in databases such as PubMed, Web of Science, and EMBASE is as follows: we will specify search terms, including combinations of “non-coding RNA”, “HIF-1α”, and “colorectal cancer”, along with a date range for the literature search (for example, from 2000 to 2025) to capture the most relevant and up-to-date research.

## 1. Introduction

### 1.1. Non-Coding RNAs

Approximately 75 percent of the human genome undergoes transcription into RNA, in contrast to the mere 3 percent that is transcribed into protein-coding mRNAs, as documented in the literature [1]. The class of non-coding RNAs (ncRNAs) encompasses long ncRNAs (lncRNAs), circular RNAs (circRNAs), microRNAs (miRNAs), and piwi-interacting RNAs (piRNAs) [2]. MicroRNAs are small non-coding RNAs, typically 20–22 nucleotides in length, that regulate gene expression post-transcriptionally. They bind to the 3′ untranslated regions (3′UTRs) of target mRNAs, usually leading to mRNA degradation or translational repression [3,4]. Long ncRNAs are longer non-coding transcripts, often more than 200 nucleotides. They can act at various levels of gene regulation, including chromatin remodeling, transcriptional regulation, and post-transcriptional regulation [5,6]. Circular RNAs are covalently closed circular molecules formed by back-splicing events. They can function as miRNA sponges, sequestering miRNAs and preventing their interaction with target mRNAs [7,8].

Extensive bodies of evidence have highlighted the pivotal roles played by ncRNAs in human malignancies, with these insights actively being translated into clinical practice. While the specific biological functions of certain ncRNAs are still being unraveled, a compelling theme has emerged: ncRNAs constitute a hierarchical, higher-order gene regulatory domain, with the potential to theoretically modulate the expression of numerous downstream mRNA targets. Notably, some ncRNAs exhibit remarkable stability, persisting in the bloodstream and presenting opportunities for the highly accurate and sensitive screening of major human cancers using minimal blood samples [9,10]. Furthermore, ncRNAs can serve as therapeutic targets, with delivery strategies drawing upon established knowledge regarding the delivery of RNAi and oligonucleotides targeting protein-coding mRNAs [11]. The identification and functional annotation of CDR1as [8], in conjunction with the initial profiling of circRNAs [7], sparked a surge in circRNA research. Accumulating evidence underscores the significant roles played by circRNAs in various diseases, including cancers [12,13,14,15,16]. Recently, multiple circRNAs have been found to be overexpressed in colorectal cancer (CRC) and to facilitate proliferation, migration, and metastasis through the upregulation of HIF-1α [17,18,19,20]. In addition to circRNAs, miRNAs and lncRNAs are relatively newer entities within the ncRNA landscape and are less well characterized. However, due to their prevalent occurrence, specific expression patterns, functionally significant roles in diseases, and potential clinical applications, they are garnering substantial attention [21,22]. This review will compile existing research findings and discuss the specific mechanisms of ncRNA and HIF-1α interactions, along with their potential applications in CRC treatment.

### 1.2. HIF-1α

**Figure 1:**
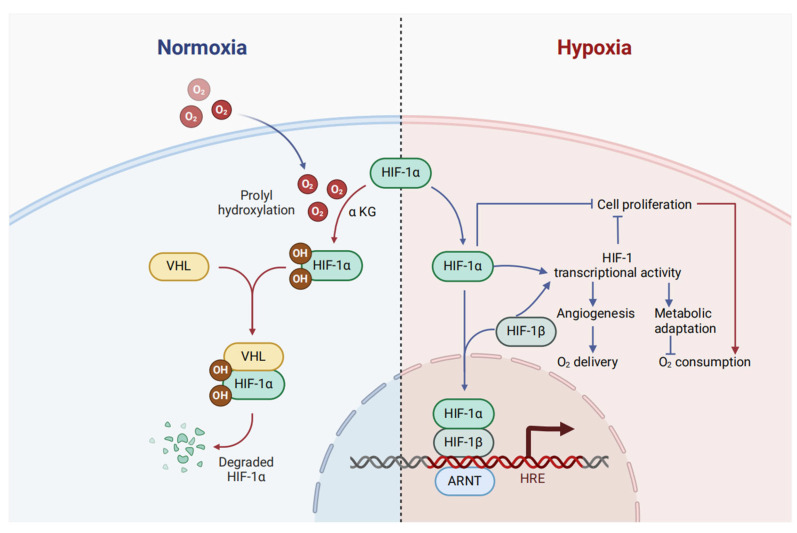
The HIF-1 transcription factor regulates the balance between oxygen supply and utilization. In environments rich in oxygen, PHD domain-containing proteins harness O2 and α-ketoglutarate to catalyze the hydroxylation of HIF-1α, subsequent to which the protein interacts with VHL, becomes ubiquitinated, and is then degraded via the proteasomal machinery. By contrast, during hypoxic conditions, hydroxylation is suppressed, culminating in an increase in HIF-1α levels. Subsequently, HIF-1α can either exert direct effects on cell proliferation or form a heterodimer with HIF-1β, thereby activating the expression of a diverse array of target genes. A significant proportion of these genes encode enzymes and transporters that are pivotal in regulating cellular metabolism. The red and blue arrows represent pathways that are predominant under normoxic and hypoxic conditions, respectively.

The microenvironment of solid tumors commonly exhibits hypoxia. This condition is a consequence of the rapid and unregulated growth of tumor cells, which leads to restricted oxygen supply [23]. Hypoxia-inducible factor 1 (HIF-1) plays a pivotal role in the regulation of hypoxic responses. Elevated levels of HIF-1 are frequently linked to poor prognoses in diverse cancer types [24]. Structurally, HIF-1 is a heterodimeric complex composed of a constantly active HIF-1β subunit and an oxygen-sensitive HIF-1α subunit [25,26]. This complex can recognize the consensus sequence 5′-RCGTG-3′, which is either present within or close to genes under the control of HIF-1 [27]. The stability of HIF-1α is regulated in an oxygen-dependent manner through prolyl hydroxylation. This process promotes the association between HIF-1α and the von Hippel–Lindau tumor suppressor protein (VHL), a crucial part of an E3 ubiquitin ligase. Such an interaction triggers the ubiquitination and subsequent degradation of HIF-1α via the proteasome [28] (Figure 1). Furthermore, the stability of HIF-1α is influenced by the cell’s metabolic state. Alpha-ketoglutarate, a metabolite of the tricarboxylic acid cycle, acts as a substrate for prolyl hydroxylases in the presence of oxygen. These enzymes incorporate one oxygen atom into a proline residue (Pro-564 or Pro-403 in human HIF-1α specifically) and the other oxygen atom into α-ketoglutarate, transforming it into succinate and carbon dioxide (Figure 1). HIF-1α controls numerous adaptive responses to hypoxia, such as cell proliferation, metabolic changes, and angiogenesis. The transcriptional activity of the HIF-1α/HIF-1β heterodimer regulates angiogenesis and metabolic reprogramming. By contrast, cell proliferation is regulated by a combination of transcriptional and non-transcriptional processes mediated by HIF-1α [29].

HIF-1α serves as a pivotal regulator in the spectrum of cancer-related processes, encompassing metabolic reprogramming, angiogenesis, metastasis, and resistance to apoptosis. Specifically, in metabolic reprogramming, HIF-1α orchestrates the hypoxia-driven metabolic alterations in tumor cells, impacting both glycolysis and lipid metabolism [30,31,32]. In the realm of angiogenesis, HIF-1α stimulates VEGF production, thereby enhancing vascular density and reducing oxygen diffusion distance, ultimately disrupting local blood flow dynamics within the tumor microenvironment [33]. Concerning resistance to apoptosis, HIF-1α acts to inhibit apoptotic pathways, promoting the adaptive survival of cancer cells [34]. Regarding metastasis, HIF-1α exerts its influence by directly or indirectly modulating the expression of key genes at various stages of the metastatic cascade [35,36].

### 1.3. Colorectal Cancer

Variability in CRC prevalence across nations is attributed to multiple contributing factors, including socio-economic status, with lower socio-economic levels correlating with heightened CRC susceptibility. Advancements in screening, early interventions, and improved therapeutic strategies have led to a reduction in CRC mortality by approximately 35% from 1990 to 2007, with a further decrease of around 50% from peak mortality rates [37]. Nonetheless, it must be recognized that the overall decline in colorectal cancer (CRC) mortality might have concealed the mortality trend among young adult CRC patients. Data from the SEER database indicate that although the mortality rate for young adults (aged 20–54 years, white) dropped from 6.3 per 100,000 in 1970 to 3.9 in 2004, it increased to 4.3 per 100,000 in 2014.

While progress in evidence-based therapies and early diagnostic strategies for colorectal cancer is commendable, substantial knowledge deficits persist. These gaps necessitate further investigation to enhance our comprehension of the disease’s pathogenesis, to refine therapeutic efficacy, and to extend patient lifespan without compromising their quality of life. Emerging research has unveiled a complex and functional landscape of ncRNAs in CRC [38]. Numerous ncRNAs exhibit differential expression across normal tissue, primary, and metastatic castration-resistant CRCs, with some capable of independently promoting proliferation [39,40,41]. These individual ncRNAs often carry prognostic implications. Furthermore, ncRNAs exhibit enhanced stability when contrasted to their linear forms and are extensively present in human plasma exosomes, positioning them as prime candidates for minimally or non-invasive biomarkers [42,43]. Of greater significance, ncRNAs are capable of engaging with the hypoxic tumor microenvironment, a hallmark of solid tumors, particularly with HIF-1α, to synergistically modulate the progression of colorectal cancer [44,45].

In 2023, an interesting review on the interactions between ncRNAs and HIF-1α in cancer was published in the European Journal of Pharmacology. This article precisely focuses on the context of colorectal cancer, delving deep into the interactions between these ncRNAs and HIF-1α [39]. It particularly emphasizes highlighting the clinical relevance of HIF-1α-related ncRNAs in CRC, aiming to bridge the existing gap between the results obtained from basic research and the clinical implementation of HIF-1α-related ncRNAs as biomarkers for diagnosis, prognosis, and treatment in CRC. Moreover, this review centers around the core theme that, in future large-scale clinical trials, it is necessary to comprehensively evaluate the therapeutic potential and safety of HIF-1α-related ncRNAs.

## 2. HIF-1α Interacting ncRNAs in Colorectal Cancer

### 2.1. Long ncRNA

Previously regarded as transcriptional noise, lncRNAs have emerged as a ubiquitous class of ncRNAs with significant biological functions over the past decade [46]. Despite their lower abundance among ncRNAs, the context-specific expression patterns and the altered transcriptome levels of lncRNAs in CRC contexts render them viable candidates for biomarkers. A comprehensive study employing whole-transcriptome sequencing of 10 CRC and para-cancerous specimens identified 2852 lncRNA transcripts that displayed dysregulation when compared to normal control samples [47]. Research has shown that lncRNAs can modulate gene expression through various mechanisms and perform several common functions. In the realm of epigenetics, circRNAs can recruit a range of epigenetic factors to enhance signal transduction and transcription [48]. At the transcriptional level, lncRNAs interact with gene promoters, transcription factors (TFs), and co-factors to regulate transcription [49]. At the post-transcriptional level, lncRNAs engage with multiple splicing factors, such as miRNAs and RNA-binding proteins (RBPs), to influence the precursor mRNAs of numerous genes [50]. Moreover, lncRNAs regulate a wide array of RNA and protein modifications, thereby affecting their activity and stability [51]. Notably, peptides derived from lncRNAs exert pivotal functions within a multitude of biological pathways [52].

**Figure 2:**
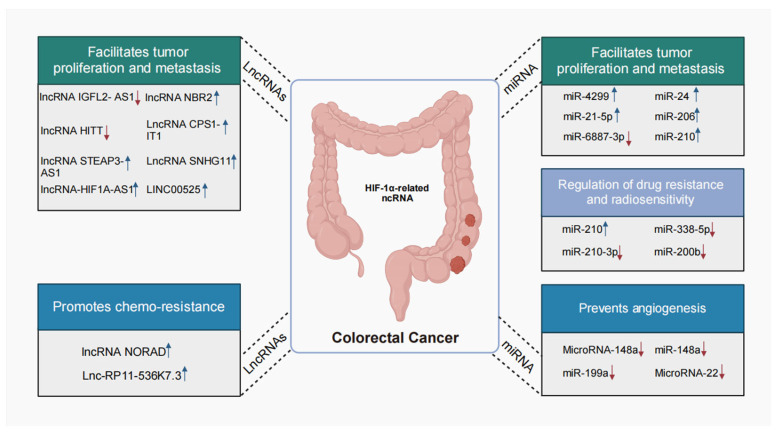
The emerging roles of HIF-1α-related lncRNAs and miRNAs in colorectal cancer. HIF-1α-related lncRNAs and miRNAs execute a variety of functions in CRC, such as proliferation, metastasis, chemoresistance, radiosensitivity, drug resistance, and angiogenesis. Upregulated lncRNAs are denoted by the blue arrow, while downregulated lncRNAs are marked by the red arrow.

In the past decade, an extensive body of research has revealed the functional contributions of diverse individual lncRNAs in CRC, achieved through their engagement with HIF-1α, leveraging both cellular and animal model systems [39,44,45,46,47,48,49,50,51,52,53,54,55,56,57] (Table 1). One investigation highlights the hypoxic induction of HIF-1α, which leads to the transcriptional augmentation of lncRNA STEAP3-AS1, competing with YTHDF2 for binding and thereby increasing the mRNA stability of STEAP3, which in turn enhances STEAP3 protein levels. This upregulation of STEAP3 results in the generation of cellular Fe^2+^, activating the phosphorylation of Ser 9 and inactivating GSK3β, facilitating β-catenin nuclear translocation and the subsequent activation of Wnt signaling, thereby facilitating CRC progression [58]. A plethora of studies have also demonstrated that the HIF-1α/lncRNA interface is pivotal in CRC, modulating chemotherapy responsiveness (Figure 2). For example, lnc-RP11-536 K7.3 is linked to oxaliplatin resistance and poor prognosis. The deletion of lnc-RP11-536 K7.3 suppresses proliferation, glycolysis, and angiogenesis, and improves chemosensitivity in both in vitro chemo-resistant organoids and in vivo CC cells. Moreover, lnc-RP11-536 K7.3 recruits SOX2, which then promotes the transactivation of USP7 mRNA. This process results in the deubiquitination and stabilization of HIF-1α, ultimately playing a role in the development of oxaliplatin resistance [59]. Similarly, the knockdown of NORAD might reduce hypoxia-induced malignancy in colorectal cancer. It achieves this by impeding VM and chemoresistance. The underlying mechanism involves sequestering miR-495-3p/HIF-1α, which in turn regulates EMT [60]. Moreover, LINC00511 and HITT stimulate CRC cell proliferation and inhibit apoptosis. Notably, a robust association between HIF-1α mRNA and HITT has been substantiated in human colon cancer tissues [61,62]. Significantly, elevated serum levels of lncRNA/HIF1A-AS1 have emerged as a novel prognostic biomarker for unfavorable clinical outcomes [63]. In recent investigations, the significant functions of lncRNAs in the progression of CRC and the development of chemoresistance have been revealed. Notably, these functions are often mediated through interactions with HIF-1α. This research underscores the potential of lncRNAs as innovative diagnostic and prognostic biomarkers, as well as promising therapeutic targets.

### 2.2. MicroRNAs

MiRNAs are compact, evolutionarily conserved ncRNAs with a length of roughly 22 nucleotides, which are pivotal in the modulatory processes of gene expression [79]. The first miRNA, lin4, was identified from Caenorhabditis elegans in 1993 [80]. In 2000, Reinhart et al. reported the first mammalian miRNA, let-7, which was found to suppress the expression of the heterochronic gene lin-41 by means of sequence-specific RNA–RNA interactions with the 3′-UTR of its mRNA [81,82]. In 2002, Dr. Calin’s research team was the first to establish the role of miRNAs in human cancer development, revealing that miR-16-1 and miR-15a, situated on chromosome 13q14, are frequently deleted in B-cell chronic lymphocytic leukemia (CLL) [83]. Over the last decade, a multitude of investigations have clarified the roles of miRNAs in a variety of pathologies, with a particular emphasis on cancer. A transcriptomic study of plasma from 1382 CRC patients has highlighted the potential integration of plasma miRNAs into fecal immunochemical test-based CRC screening protocols. This integration aims to optimize colonoscopy scheduling by focusing on individuals most likely to benefit significantly from the procedure [84]. The regulation of gene expression by miRNAs is a universal feature among all recognized cancer cell types. Throughout tumor development, miRNAs exhibit distinct biological characteristics, suggesting their potential for cancer classification and improved prognosis.

Interactions between HIF-1α and miRNAs collectively influence the progression of CRC, including aspects of drug resistance and chemosensitivity (Figure 2 and Table 1). CRC cells exhibit enhanced exosome production, and the miRNAs contained within these exosomes play a significant role in disease progression, potentially facilitating metastasis. For instance, exosomes derived from hypoxic conditions promote the proliferation and metastasis of CRC by exporting HIF-1α-induced miR-4299 and regulating its target gene ZBTB4 [65]. Additionally, hypoxic repression of miR-338-5p stabilizes a HIF-1α-driven feedback loop via direct targeting of IL-6/STAT3/Bcl2 signaling, driving chemoresistance in colorectal cancer. Preclinical models demonstrate that combinatorial miR-338-5p restoration and HIF-1α inhibition disrupt this axis to re-sensitize tumors to oxaliplatin [73]. In CRC, miR-20a is significantly downregulated under hypoxic conditions. Overexpression of miR-20a has been found to mitigate hypoxia-induced autophagy. Mechanistically, miR-20a inhibits the hypoxia-induced autophagic flux by targeting multiple key autophagy regulators, such as ATG5 and FIP200. Through dual-luciferase assays, it has been demonstrated that miR-20a directly binds to the 3′-untranslated region of ATG5 and FIP200 mRNAs, thereby regulating their mRNA and protein levels [85]. Another study in this type of cancer has shown the HIF-1α induces an elevation in the levels of miR-361-3p within hypoxic extracellular vesicles (EVs) in the context of CRC. The upregulated miR-361-3p in CRC exerts its effects by directly targeting TNF receptor-associated factor 3. This targeting event inhibits cell apoptosis and promotes cell growth, ultimately leading to the activation of the noncanonical NF-κB pathway [86]. Importantly, the presence of these miRNAs in body fluids, derived from exosomes, underscores their substantial value as diagnostic markers for CRC.

Angiogenesis constitutes a pivotal factor in tumor progression. The importance of this process, encompassing the generation of novel blood vessels, in the expansion of solid neoplasms is extensively recognized. MicroRNAs have been shown to play a crucial role in the progression of CRC by modulating angiogenesis through interactions with HIF-1α. The microRNA miR-148a markedly inhibits angiogenesis by suppressing VEGF through downregulating the PERK/HIF-1α/VEGF axis, potentially leading to the inhibition of angiogenesis. Conversely, the downregulation of miR-148a is associated with an increased risk of early recurrence in CRC [72]. Furthermore, miR-148a represses HIF-1α/VEGF and Mcl-1 by directly targeting ROCK1/c-Met, thereby diminishing angiogenesis and enhancing apoptosis in colon cancer cells. Notably, patients with metastatic CRC (mCRC) displaying elevated levels of serum miR-148a demonstrate a more favorable therapeutic response to the combination of chemotherapy and bevacizumab (standard dose; 5 mg/kg) compared to those without such overexpression [68]. The microRNAs miR-206, miR-22, and miR-199a may exert anti-angiogenic effects in colon cancer [74,77,78]. Beyond angiogenesis, the interaction between miRNAs and HIF-1α also promotes glycolysis, thereby advancing the progression of CRC. The HIF-1α-induced miR-23a∼27a∼24 cluster (comprising miR-24, miR-27a, and miR-23a) is a critical regulator that redirects CRC metabolism from oxidative phosphorylation to glycolysis and, by controlling their expression, holds promise for the suppression of CRC progression [70].

Accumulating studies indicate that miRNAs are central to the molecular underpinnings of drug resistance and radiosensitivity through interactions with HIF-1α. Research has proposed that the suppression of miR-210-3p subsequent to 5-FU administration maintains DNA damage repair and metabolic adaptations, thereby mitigating the effects of the drug [71]. Additionally, HIF-1α activates miRNA-210, which potentiates autophagy and diminishes radiation sensitivity by repressing Bcl-2 expression in colon cancer cells [76]. A further investigation has unveiled an HIF-1α/miR-338-5p/IL-6 feedback circuitry that is essential for hypoxic-induced resistance to chemotherapy in CRC [73]. Furthermore, miR-6887-3p and miR-200b can also influence the invasion and proliferation of CRC cells by interacting with HIF-1α [69,75].

The interaction of HIF-1α with miRNAs is pivotal in the evolution of CRC, modulating therapeutic resistance and responsiveness, stimulating angiogenesis, and orchestrating metabolic cascades. CRC cells augment the secretion of exosomes, within which miRNAs are integral to the advancement of the disease, and may further facilitate metastatic dissemination. Collectively, the intricate web of interactions involving HIF-1α and a constellation of miRNAs profoundly influences CRC at multiple biological dimensions, encompassing proliferation, dissemination, angiogenesis, metabolism, and therapeutic outcomes. This elucidation offers novel insights and potential therapeutic avenues for CRC diagnosis and intervention.

## 3. New Areas of ncRNA Research in Colorectal Cancer

Given the significant heterogeneity characteristic of colorectal cancer, it is imperative to investigate cell-type-specific ncRNAs within the tumor microenvironment. Following the pioneering identification of non-coding RNAs over three decades ago, recent technological breakthroughs have facilitated the exploration of critical dimensions of non-coding RNA research that were hitherto inaccessible.

### 3.1. RNA Vaccines Based on ncRNA

The remarkable effectiveness of SARS-CoV-2 mRNA vaccines in the fight against the COVID-19 pandemic has rekindled interest in RNA-based immunotherapies. Drew Weissman and Katalin Karikó were awarded the 2023 Nobel Prize in Physiology or Medicine for their groundbreaking work on nucleoside base editing [87]. This discovery has been crucial in facilitating the development of revolutionary vaccines. An important advantage of circRNA lies in its covalently closed-loop structure. This structure protects circRNA from nuclease-mediated degradation, thereby improving its stability and persistence. Hypothetically, circRNAs might achieve enhanced therapeutic effects even when administered at low dosages [88]. Chen et al.’s team achieved a significant increase in protein yield from cyclic RNA translation through optimized designs, enabling efficient and sustained in vivo protein production [89]. Furthermore, RNA therapies based on miRNAs and lncRNAs have garnered considerable attention [90,91]. For instance, a research endeavor has engineered a bio-scaffold that incorporates biomimetic nanosystems designed to target the lncRNA Pvt1. This scaffold is instrumental in orchestrating a tri-functional immune response aimed at preventing the postoperative recurrence of CRC. This scaffold elicits a vigorous immune memory, effectively quelling ectopic tumor rechallenges and concurrent metastatic dissemination (with a success rate of 100%). Furthermore, the bio-scaffold demonstrates a 70.8% decrease in synchronous distant metastasis [92]. Notably, the number of ncRNA-based therapies undergoing clinical trials for various genetic, metabolic, and oncological conditions is steadily growing.

### 3.2. Non-Coding RNA and Disulfidptosis

The recently characterized phenomenon of disulfidptosis, a caspase-independent mode of programmed cell death, constitutes a novel frontier in cancer research, driven by its pathogenesis rooted in disulfide stress. Its relevance in the realm of cancer biology is underpinned by two primary considerations [93]. Initially, it embodies a unique mode of regulated cell death, distinct from other known forms [94]. Secondly, it holds promise as a potent therapeutic target for future cancer treatments. In a study by Dong et al., TCGA datasets were meticulously analyzed to construct a prognostic disulfidptosis-related lncRNA signature for colon cancer. Four lncRNAs were pinpointed as the most prognostically relevant indicators for patient survival, with a notable survival advantage observed in the low-risk subgroup compared to the high-risk subgroup. Bioinformatics analysis revealed distinct metabolic pathways and immune profiles. The model was further validated using independent datasets, and the differential expression of the lncRNAs was confirmed through rt-qPCR in tissue and cellular samples [95]. These findings underscore the importance of understanding the roles of various ncRNAs in disulfidptosis, providing crucial insights into their potential mechanisms of action.

### 3.3. Single Cell and Spatial Transcriptomics

Non-coding RNA expression is characterized by its specificity and stringent regulation. Within the context of cancer, a plethora of lncRNAs and miRNAs display expression profiles that are distinct to specific tumor types or subtypes [96]. While bulk transcriptomic analyses typically reveal low ncRNA expression, recent research has detected elevated expression in specific cellular subpopulations [97], presenting opportunities to explore intratumoral heterogeneity. Consequently, the examination of ncRNA expression signatures at the single-cell resolution or across spatially distinct tumor compartments could reveal novel molecular subtypes or infrequent cell populations within prostate cancer, thereby providing valuable insights into tumor complexity.

Traditional cancer research has primarily centered on detailed molecular and clinical investigations of key pathways and genes. In recent years, advancements in high-throughput technologies have significantly accelerated the collection of extensive cancer genomics data, encompassing genomics, transcriptomics, epigenomics, and other genomic fields, with the goal of thoroughly and systematically assessing various aspects of tumor characteristics [98]. As histological data rapidly expand, numerous bioinformatics databases and tools have been developed, greatly aiding in the storage, efficient retrieval, comprehensive integration, and in-depth analysis of these data [99]. The integration and detailed analysis of these datasets have fundamentally transformed the cancer research landscape, offering deeper insights into the molecular mechanisms driving tumorigenesis, elucidating the tumor microenvironment (TME) characteristics, accurately identifying potential biomarkers for early detection and prognostic assessment, and guiding the development of precision-targeted therapies for individual patients [100]. For instance, an investigation elucidated the involvement of TM4SF1 in facilitating EMT and cancer stem cell characteristics in CRC through the Wnt/β-catenin/SOX2 signaling axis, leveraging data resources from The Cancer Genome Atlas and the Gene Expression Omnibus repositories [101]. This approach significantly enhances the efficiency and accuracy of research.

## 4. Clinical Relevance of HIF-1α-Related ncRNAs in Colorectal Cancer

**Figure 3:**
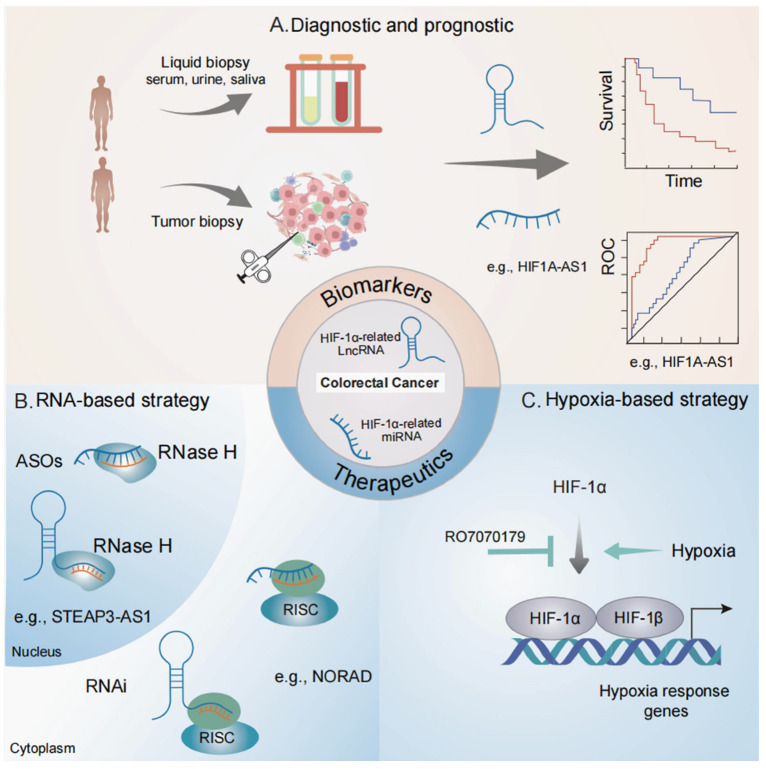
Potential applications of HIF-1α-associated lncRNAs and miRNAs as biomarkers and therapeutic targets in colorectal cancer. (**A**) Diagnostic and prognostic potential of HIF-1α-associated lncRNAs and miRNAs. These ncRNAs can be consistently detected in various biospecimens, including both traditional tumor biopsy samples and liquid biopsy samples (such as blood and urine). (**B**) Therapeutic potential of HIF-1α-associated lncRNAs and miRNAs. Modulating the expression and function of HIF-1α-associated lncRNAs and miRNAs through RNAi and ASO therapies, specifically targeting the cytoplasm and nucleus, respectively. (**C**) Inhibitors effectively regulate the expression and function of HIF-1α. The HIF-1α mRNA antagonist RO7070179 suppresses the expression of hypoxia response genes.

Many ncRNAs associated with HIF-1α have been found to be dysregulated in CRC. Moreover, these ncRNAs exhibit high specificity and are readily identifiable across a spectrum of biological matrices, including tissues, serum, saliva, urine, and plasma. Consequently, HIF-1α-associated ncRNAs hold significant promise as both diagnostic and prognostic biomarkers, as well as potential therapeutic endpoints, within the context of CRC (Figure 3).

### 4.1. Biomarker Potentials

The serum levels of HIF1A-AS1 were significantly higher in CRC patients compared to healthy controls (*p* < 0.05). An ROC curve analysis indicated that HIF1A-AS1 has a high diagnostic potential for differentiating CRC patients from healthy individuals, with an AUC of 0.960. A Kaplan–Meier survival analysis revealed that factors such as differentiation grade, tumor size, TNM classification, T stage, N stage, M stage, and serum HIF1A-AS1 levels were all associated with the prognosis of CRC patients (all *p* < 0.05). Based on these findings, a serum-based biomarker panel centered on HIF1A-AS1 has been developed. This panel can be used to assess the need for rebiopsy in cases where carcinoembryonic antigen and carbohydrate antigen 19-9 levels are elevated, despite initially negative biopsies [63]. It has been reported that bioinformatic analyses of the CRC-transcriptomes indicated that the lncRNA/PVT1 locus might exert a far-reaching impact on the expression and functions of other key genes within two vital CRC-related signaling pathways, specifically the TGFβ/SMAD and Wnt/β-Catenin pathways. Furthermore, PVT1 acts as a novel oncogenic enhancer of MYC, with its activity being epigenetically regulated through aberrant methylation in CRC. The findings also suggested that the expression of PVT1/lncRNA could be a promising prognostic biomarker and a potential therapeutic target in colorectal cancer [102]. In addition to HIF1A-AS1, several other lncRNAs, including lncRNA HITT, CircMYH9, and miR-206, show promise as candidates for diagnostic and prognostic biomarkers in CRC [18,19,20,21,22,23,24,25,26,27,28,29,30,31,32,33,34,35,36,37,38,39,40,41,42,43,44,45,46,47,48,49,50,51,52,53,54,55,56,57,58,59,60,61,62,63,64,65,68,69,70,71,72,73,74,75,76,77,78,79,80,81,82,83,84,85,86].

Currently, several ongoing clinical trials are recruiting subjects to evaluate the value of ncRNAs as diagnostic and prognostic markers in CRC (Table 2). The research on HIF-1α-associated lncRNAs and miRNAs shows great promise for future exploration. Consequently, hypoxia-responsive lncRNAs and miRNAs are becoming increasingly attractive as potential cancer biomarkers for both diagnosis and prognosis (Figure 3). For instance, a clinical trial (NCT04269746) is now examining the potential of lncRNA CCAT1 as a CRC biomarker. The area of liquid biopsies is attracting significant attention. Liquid biopsies are less invasive than traditional tissue biopsies and are highly suitable for continuous monitoring of the disease. Due to their unique expression profiles, lncRNAs are emerging as feasible candidates for liquid biopsy-based biomarkers, with preclinical testing having been carried out in plasma (NCT04269746 and NCT06307249). In metastatic CRC [72], exosomes offer another source of extracellular lncRNAs, with miR-21-5p and miR-4299 being highly expressed in exosomes derived from CRC cells [64,65,66,68,69,70,71,72,73,74,75,76,77,78,85,86].

### 4.2. Therapeutic Potential in Targeting HIF Signaling

The hypoxia condition, characterized by its pivotal involvement in the onset, advancement, dissemination, and therapeutic resistance of neoplasms, has garnered attention as a highly attractive therapeutic niche [103]. Among the numerous avenues of investigation, the modulation of HIF signaling stands out as a focal point. The tumor’s adaptive response to hypoxia is predominantly governed by HIF signaling, which is initiated through a meticulously controlled signaling cascade. Each component of this pathway presents a potential target, encompassing the suppression of HIF-α protein aggregation with PHD inhibitors [104,105], the inhibition of PI3K/mTOR [106,107], the targeting of topoisomerases [108,109], the interference with microtubule dynamics, the disruption of thioredoxin activity [110], the attenuation of HSP90 [111], and the inhibition of HDACs [112]. Moreover, inhibitors that prevent the HIF-α–HIF-β heterodimerization, such as PAS domain inhibitors [113,114], those that interfere with HIF–HRE interaction, including HRE inhibitors [115,116], and those that disrupt coactivator engagement, such as TAD inhibitors, are also being explored [117,118]. At present, multiple clinical trials are underway to explore hypoxia-targeting strategies in cancer using investigational drugs. Significantly, a clinical investigation (NCT02564614) is currently evaluating the impacts of the HIF-1α mRNA antagonist RO7070179 in adult liver cancer patients. Additionally, a phase 3 clinical trial (NCT04195750) is assessing the effectiveness of the HIF-2α inhibitor MK-6482 in patients diagnosed with advanced renal clear cell carcinoma. A more in-depth comprehension of the regulatory mechanisms of HIF signaling pathways will contribute to the development of innovative drugs for targeting hypoxic tumors.

### 4.3. Therapeutic Potential in Targeting ncRNAs

Inhibiting HIF-1α and HIF-2α is a potential cancer treatment strategy. However, resistance is a common challenge. For example, extended use of PT2399, a specific HIF-2 inhibitor, can lead to resistance characterized by increased tumor blood vessel formation and higher VEGF levels [119]. One study has found that lncRNA, NORAD, and HIF-1α are elevated in CRC tissues and positively associated. The NORAD knockdown reduces hypoxia-induced VM formation, 5-FU resistance, and HIF-1α-mediated EMT by ponging miR-495-3p, suggesting NORAD as a potential therapeutic target for CRC [60]. The downregulation of miR-210-3p empowers resistant cells to mitigate the toxic impact of the drug. This is achieved by upregulating the expression of the RAD-52 protein, which is accountable for DNA damage repair. Additionally, the downregulation of miR-210-3p augments oxidative phosphorylation (OXPHOS). It performs as such by elevating the expression levels of succinate dehydrogenase subunits D, reducing intracellular succinate levels, and suppressing HIF-1α expression. Collectively, these adaptive changes result in enhanced cell survival upon drug exposure [71].Therefore, the development of novel and more efficacious treatment modalities is of great significance for enhancing clinical outcomes. One feasible approach to counteract hypoxia-induced cancer invasion is to target the downstream elements of the HIF pathway. The association between hypoxia-related ncRNAs and cancer progression indicates their potential as therapeutic targets. As a result, precisely targeting ncRNAs within the hypoxic TME presents a promising strategy for the treatment of diverse and complex cancer types.

The employment of efficient gene modulation techniques, such as overexpression or knockdown, can augment the precision of targeting hypoxia-associated lncRNAs and circRNAs for therapeutic purposes (Figure 3). Currently, RNA-based therapeutic strategies predominantly rely on RNAi and antisense oligonucleotides (ASOs) to specifically target particular RNA segments and molecules. Investigations in murine models have shown that the utilization of ASOs and RNAi to target oncogenic hypoxia-related lncRNAs and circRNAs can efficiently inhibit tumor growth. Non-coding RNAs that display substantial oncogenic potential in CRC emerge as attractive candidates for innovative therapeutic approaches. In preclinical investigations, lncRNAs like STEAP3-AS1, NORAD, and LINC00511 have been discovered to facilitate CRC cell growth and malignancy [58,59,60,61]. Additionally, the lnc-RP11-536 K7.3 is associated with the chemosensitivity of CRC [59]. MicroRNAs, like miR-148a, reduce angiogenesis and enhance apoptosis in colon cancer cells [72]. Considering their indispensable roles in pathogenesis, these oncogenic ncRNAs represent a compelling focus for the development of novel therapeutic strategies.

## 5. Concluding Remarks and Perspectives

The high mortality rate of CRC is attributed to its rapid progression and the absence of early diagnostic markers. Therefore, an imperative requirement exists for the discovery of novel tumor biomarkers and therapeutic endpoints to facilitate early diagnosis, precise prognostication, and efficacious therapeutic interventions. The altered expression of HIF-1α-related ncRNAs in tumor samples, coupled with their easy detection in bodily fluids, makes them promising candidates for non-invasive liquid biopsy approaches. Furthermore, these HIF-1α-related ncRNAs have been shown to significantly impact various cancer hallmarks, including tumor metabolism, immune evasion, angiogenesis, migration/invasion, and growth/proliferation. Consequently, HIF-1α-related ncRNAs hold potential as biomarkers or therapeutic targets in CRC.

Notwithstanding the remarkable progress achieved in elucidating the functions of ncRNAs within the hypoxic TME, numerous challenges and limitations persist and demand resolution. Moreover, several key questions remain unanswered. (i) When targeting HIF-1α-associated ncRNAs in cancer, how can we safeguard against affecting the expression of essential genes in normal tissues? The identification of target ligands characterized by high affinity and stability might be beneficial in minimizing potential side effects and toxicity. Additionally, the development of refined delivery systems tailored for the accurate targeting of circRNAs is of utmost importance. (ii) What are the factors responsible for the disparities in hypoxic responses witnessed between cell lines and animal models? The majority of current studies have predominantly concentrated on two-dimensional cell culture models. As such, future research endeavors should integrate three-dimensional tumor organ models that incorporate the TME to gain more comprehensive insights [120]. (iii) Is there an augmentation of therapeutic efficacy when HIF-1α-associated ncRNA-targeting therapies are combined with other anti-tumor approaches? A recent investigation demonstrated that the combination of ASO-induced downregulation of lncRNAs and traditional chemotherapeutic drugs was more efficacious than chemotherapy alone in the treatment of CRC [64]. Consequently, the integration of therapies that target HIF-1α-associated ncRNAs with other anti-cancer modalities is likely to enhance treatment effectiveness. (iv) The utilization of ncRNAs within bodily fluids as prospective biomarkers for cancer diagnostics and prognostication presents a formidable challenge. Recent progress in RNA analytical methodologies has enabled the creation of innovative platforms, which leverage machine learning algorithms to discern complex or composite biomarker profiles [121]. Notably, circRNAs, known for their stability and high translation efficiency, have attracted considerable attention [122].

Notably, a divergence exists between the results obtained from basic research and the clinical implementation of HIF-1α-related ncRNAs as diagnostic, prognostic, and therapeutic biomarkers. As a result, in future large-scale clinical trials, it is essential to comprehensively evaluate the therapeutic potential and safety of HIF-1α-related ncRNAs.

## Figures and Tables

**Table 1:** Summary of HIF-1α-related lncRNAs and miRNAs in CRC.

NcRNAs	Expression	Target Genes	Functions	Cancers	Reference
lncRNA IGFL2- AS1	Upregulate	HIF-1α	Promotes CRC cell proliferation and invasion	CRC	[54]
lncRNA NBR2	Downregulate	HIF-1α	Suppresses the progression	CRC	[55]
LINC00525	Upregulate	HIF-1α	Regulates the Warburg effect	CRC	[44]
lncRNA NORAD	Upregulate	HIF-1α	Enhances vasculogenic mimicry and contributes to resistance against 5-fluorouracil	CRC	[60]
lncRNA STEAP3-AS1	Upregulate	STEAP3	Facilitates CRC progression by inhibiting m(6)A-mediated STEAP3 mRNA degradation.	CRC	[58]
Lnc-RP11-536 K7.3	Upregulate	HIF-1α	Enhances progression and chemoresistance via the SOX2/USP7/HIF-1α signaling pathway.	CRC	[59]
LncRNA SNHG11	Upregulate	TCEB1	Facilitates metastasis through the downregulation of TCEB1 mRNA.	CRC	[57]
lncRNA HITT	Downregulate	HIF-1α	Inhibits cancer cell adaptation to hypoxia by collaborating with Ezh2 to repress HIF-1α transcription.	CRC	[64]
LncRNA CPS1-IT1	Downregulate	HIF-1α	Inhibits EMT and metastasis by suppressing autophagy through the inactivation of HIF-1α.	CRC	[53]
lncRNA/HIF1A-AS1	Upregulate		Facilitates tumor proliferation and metastasis	CRC	[63]
miR-4299	Upregulate	ZBTB4	Enhances the proliferation and metastasis by modulating the HIF-1α/miR-4299/ZBTB4 pathway	CRC	[65]
miR-21-5p	Upregulate	HIF-1α	Facilitates the proliferation and migration of CRC cells	CRC	[66]
miR-210	Upregulate	P53	Induces EMT and drug resistance via activation of p53	CRC	[67]
MicroRNA-148a	Downregulate	HIF-1α	Triggers apoptosis and inhibits angiogenesis with bevacizumab by suppressing ROCK1/c-Met through HIF-1α	CRC	[68]
miR-6887-3p	Downregulate	RAP1/MAPK	Facilitates oncogenesis via the RAP1/MAPK signaling pathway and is suppressed by miR-6887-3p.	CRC	[69]
miR-24	Upregulate	HIF-1α	Promotes CRC progression via reprogramming metabolism	CRC	[70]
miR-210-3p	Downregulate	HIF-1α	Facilitates metabolic adaptation and maintains DNA damage repair in CRC cells resistant to 5-fluorouracil treatment.	CRC	[71]
miR-148a	Downregulate		Inhibits VEGF production and early relapse by targeting HIF-1α	CRC	[72]
miR-338-5p	Downregulate	IL-6	Triggers drug resistance via the HIF-1α/miR-338-5p/IL-6 feedback loop.	CRC	[73]
miR-206	Upregulate	HIF-1α	inhibits the Met/ERK/Elk-1/HIF-1α/VEGF-A pathway to prevent angiogenesis.	CRC	[74]
miR-200b	Downregulate	HIF-1α	Affects the EMT-MET plasticity	CRC	[75]
miR-210	Upregulate	Bcl-2	Decreases radiosensitivity via the HIF-1α/miR-210/Bcl-2 pathway	CRC	[76]
miR-199a	Downregulate	HIF-1α	Inhibits CRC progression via the HIF-1α/VEGF pathway.	CRC	[77]
MicroRNA-22	Downregulate	HIF-1α	Suppresses angiogenesis	CRC	[78]

**Table 2:** Example of preclinical and clinical investigations examining the exploitation of HIF-1α-related ncRNAs in CRC.

NCT Number	NcRNAs	Sample	Biomarker	Study Status	Disease	Study Type
NCT06351384	miRNA	Peripheral blood	Diagnostic	Recruiting	CRC	Observational
NCT02635087	miRNA	Postoperative specimen	Therapeutic response	Recruiting	CRC	Observational
NCT02466113	miRNA	Postoperative specimen	Therapeutic response	NA	CRC	Interventional
NCT01828918	miRNA	Peripheral blood	Diagnostic	Phase 1	CRC	Interventional
NCT04269746	lncRNA	Peripheral blood	Diagnostic	Complete	CRC	Observational
NCT06432413	lncRNA	Peripheral blood	Diagnostic	Complete	CRC	Observational
NCT06307249	lncRNA	Blood or tissue	Diagnostic	Phase 1	CRC	Interventional

## Data Availability

Not applicable.
